# Does Anybody Want an Injectable Rotavirus Vaccine, and Why? Understanding the Public Health Value Proposition of Next-Generation Rotavirus Vaccines

**DOI:** 10.3390/vaccines10020149

**Published:** 2022-01-20

**Authors:** William P. Hausdorff, Jessica Price, Frédéric Debellut, Jessica Mooney, Andrew A. Torkelson, Khatuna Giorgadze, Clint Pecenka

**Affiliations:** 1 Center for Vaccine Innovation and Access, PATH, 455 Massachusetts Ave NW, Washington, DC 20001, USA; 2 Faculty of Medicine, Université Libre de Bruxelles, 1070 Brussels, Belgium; 3 Center for Vaccine Innovation and Access, PATH, 2201 Westlake Ave, Suite 200, Seattle, WA 98121, USA; jprice@path.org (J.P.); jmooney@path.org (J.M.); cpecenka@path.org (C.P.); 4 Center for Vaccine Innovation and Access, PATH, Rue de Varembé 7, 1202 Geneva, Switzerland; fdebellut@path.org; 5 Linksbridge SPC, 808 5th Ave N, Seattle, WA 98109, USA; andy.torkelson@linksbridge.com (A.A.T.); khatuna.giorgadze@linksbridge.com (K.G.)

**Keywords:** rotavirus, gastroenteritis, product preferences, cost-effectiveness, combination vaccines, value proposition

## Abstract

Routine infant immunization with live, oral rotavirus vaccines (LORVs) has had a major impact on severe gastroenteritis disease. Nevertheless, in high morbidity and mortality settings rotavirus remains an important cause of disease, partly attributable to the sub-optimal clinical efficacy of LORVs in those settings. Regardless of the precise immunological mechanism(s) underlying the diminished efficacy, the introduction of injectable next-generation rotavirus vaccines (iNGRV), currently in clinical development, could offer a potent remedy. In addition to the potential for greater clinical efficacy, precisely how iNGRVs are delivered (multiple doses to young infants; alongside LORVs or as a booster; co-formulated with Diphtheria-Tetanus-Pertussis (DTP)-containing vaccines), their pricing, and their storage and cold chain characteristics could each have major implications on the resultant health outcomes, on cost-effectiveness as well as on product preferences by national stakeholders and healthcare providers. To better understand these implications, we critically assessed whether there is a compelling public health value proposition for iNGRVs based on potential (but still hypothetical) vaccine profiles. Our results suggest that the answer is highly dependent on the specific use cases and potential attributes of such novel vaccines. Notably, co-formulation of iNGRVs with similar or greater efficacy than LORVs with a DTP-containing vaccine, such as DTP-Hib-HepB, scored especially high on potential impact, cost-effectiveness, and strength of preference by national stakeholders and health care providers in lower and middle income countries.

## 1. Introduction

The COVID-19 pandemic represents a clear example where the development of highly effective vaccines immediately led to strong use recommendations from global and national public health agencies and rapid (though not yet optimal) uptake in the target populations where the vaccines were available. However, for many other pathogens responsible for significant mortality and morbidity, the development of a highly effective vaccine does not necessarily translate into strong policy recommendations and widespread demand, even in the countries with the highest disease burdens [1]. The repeated experience with demonstrably effective vaccines failing to result in widespread adoption has led the World Health Organization (WHO) and others to advocate for the development of critical analyses of the overall public health, economic, and societal value of any new vaccine candidate well in advance of product licensure [2,3]. Variously termed “public health value propositions”, “business or investment cases”, or “full value of vaccine assessments”, these analyses aim to inform vaccine developers, donor agencies, recommending and funding bodies, and national authorities of the potential societal value of these vaccines in the context of other, competing interventions. Ideally, they would also integrate perspectives not just from recognized international experts, but also from healthcare providers, caregivers, and potential vaccine recipients [4].

## 2. The Multiple Theoretical Advantages of Next-Generation Rotavirus Vaccines

We present here the case example of a public health value proposition for next-generation rotavirus vaccines (NGRVs). The analyses described here were designed to inform NGRV developers, funders, recommending bodies, and national authorities whether—and in what way—there may be a compelling value proposition for such vaccines. The approach taken here took into account the comprehensive list of considerations embodied in WHO’s recommendations on the outline of Public Health Value Proposition on Vaccines [5] and focused on those aspects that we deemed particularly relevant to understanding the potential public health value of NGRVs.

A major push for NGRV development comes from the observation that, while the currently used live, oral rotavirus vaccines (LORVs) have led to major decreases in diarrheal disease hospitalizations and mortality in young children, the virus nonetheless remains the single largest cause of serious diarrheal disease in many middle- and lower-income countries [6,7]. In some settings, suboptimal population-level vaccination coverage with LORV likely plays a role, but there is also clear evidence that LORVs are only moderately efficacious in high-mortality settings [8]. Several NGRV candidates in the late stages of clinical development [9,10] may help address these issues. The primary focus of this value proposition is those NGRVs that are parenterally administered (injectable NGRVs or iNGRVs) to mitigate, or bypass altogether, oral vaccine-related immune “take” issues associated with breastfeeding, malnutrition, environmental enteric dysfunction, and/or competition with other gut flora [11,12,13]. In the present analyses, the perceived value of a hypothetical iNGRV endowed with various desirable properties was compared to current LORVs under a variety of use cases. The iNGRVs were also compared to a hypothetical oral NGRV (oNGRV) whose first dose is delivered to neonates followed by two doses in young infants. The latter was chosen because Phase 2 clinical results suggest that at least one oNGRV candidate may offer higher clinical efficacy than current LORVs [14].

A major question is whether recommending bodies, such as WHO’s Scientific Advisory Group of Experts (SAGE) and national authorities, will prefer iNGRVs over existing alternatives and if so, which attributes drive this preference? Notably, iNGRVs offer the theoretical prospect of improving disease impact in low- and middle-income countries (LMICs) due both to their clinical characteristics as well as to their mode of delivery. For example, iNGRVs could have clinical efficacy superior to that of LORVs when given along the infant schedule. Alternatively, they might serve as a highly immunogenic booster dose to children [13,15] who had already received LORVs in early infancy and thereby address the apparently waning vaccine efficacy [16] and effectiveness [17] described in several high-morbidity settings. A clinical study is currently examining whether co-administration of one iNGRV candidate alongside LORVs in early infancy may serve to additively or synergistically boost overall rotavirus immunogenicity and, therefore, efficacy [18]. Furthermore, iNGRVs might not trigger intussusception, a rare side effect unfortunately seen with LORVs and particularly well documented in low-mortality settings [19]. iNGRVs also offer the theoretical advantage of being combinable with other currently delivered infant vaccines, thereby eliminating the need for separate cold chain storage and administration. Finally, it has been suggested that an iNGRV offered at a substantially lower price than current LORVs—though the precise threshold usually remains ill-defined—may stimulate adoption by lower middle-income countries and even middle-income countries (MICs) who have yet to introduce rotavirus vaccines, as well as help sustain current rotavirus vaccination programs in countries graduating from support from Gavi, the Vaccine Alliance (Gavi).

Based on the above considerations, the iNGRV value proposition was designed to answer two major questions:

*1. What would be the potential health impact and cost-effectiveness in LMICs of different use cases of an iNGRV (i.e., as a standalone or combination vaccine and possibly co-administered with LORVs), utilizing different assumptions of vaccine efficacy?*

*2. How would the different use cases affect whether national stakeholders and healthcare providers in LMICs prefer an iNGRV or the existing LORVs, and how might these preferences translate into demand forecasts for an iNGRV in Gavi and non-Gavi LMICs?*

To address these questions, the iNGRV value proposition first included preliminary analyses to define and winnow down the potential use cases and vaccine formulations to be examined in detail. These results informed the scenarios subsequently examined in a health impact and cost effectiveness model, a feasibility and acceptability study conducted in six LMICs, and an extensive demand forecasting exercise. Each of these compared iNGRVs (with plausible but hypothetical attributes and under similar use cases) to current LORVs or to a hypothetical oNGRV.

## 3. Methods

The value proposition involved six interrelated but distinct analyses, models, and studies.

(i) *Preliminary determination of the relative value of primary vs booster immunization with iNGRV.* We derived relevant information from the published modeled analyses of Burnett et al. [20] on the burden of rotavirus disease potentially unpreventable by LORVs and of Rogawski et al. [21] and Lopman et al. [22] on the potential contribution of natural immunization to the observation of waning LORV efficacy.

(ii) *Elaboration of demand estimates for IPV, Diphtheria-Tetanus-Pertussis-Hib-HepB (DTP pentavalent), and DTP-Hib-HepB-IPV (DTP hexavalent) through 2030*. We incorporated the latest SAGE recommendations for IPV and DTP-hexavalent use [23] and applied a standard demand forecasting methodology used by global health partners [24] and by Linksbridge Global Vaccine Market Model (GVMM) [25]. We assumed that the Gavi Board would approve funding for DTP-hexavalent during the 2022–2025 period (in November 2018, the Gavi Board approved in principle its support for the DTP-hexavalent). We further postulated that a country’s DTP-hexavalent adoption rate would be determined by its willingness to pay a price premium (since it is likely more expensive than DTP-pentavalent), by location of polio essential facilities, and by its own programmatic readiness. Finally, we also assumed that India would continue with DTP-pentavalent and the fractional dose IPV schedule during that time period. We further constrained DTP-hexavalent uptake by expected supply availability. Finally, based on data available in the WHO clinical trials database [26], partner intelligence and GVMM supply module [25], we assumed that by 2030 there could be 3–5 DTP-hexavalent suppliers, translating into three scenarios and 80–250 million doses, progressively easing supply constraints over time.

(iii) *Selection of key NGRV attributes.* A key input variable for all the assessments described in the value proposition was the level of clinical efficacy to be assumed for each of the rotavirus vaccines examined. While efficacy data exist for each of the three licensed LORVs currently available in LMICs, in the absence of head-to-head studies we made the simplifying assumption that they showed efficacy identical to one another and that this varies by epidemiological setting. Vaccine attributes and impact assumptions for LORVs were largely derived from publicly available information on ROTARIX^®^, ROTAVAC^®^, and ROTASIIL^®^ [27]. Vaccine attributes for iNGRV were largely modeled on those projected for trivalent P2-VP8 [10], the most advanced iNGRV candidate, which is currently being evaluated in three African countries for its clinical efficacy relative to ROTARIX [28]. For purposes of the analyses discussed here, the iNGRV’s efficacy was assumed to be identical to that of the LORVs, or alternatively, arbitrarily set to prevent approximately 50% more disease. Vaccine attributes for oNGRV were modeled on those for RV3-BB, the most advanced oNGRV candidate, and most notably include delivery of the first dose to neonates followed by two doses in young infancy, with efficacy values assumed up to 50% greater than current LORVs based on Phase 2 clinical trial data [14].

Pricing information for each of the three LORVs was obtained from publicly available sources and stratified per country based on a number of factors, most notably its Gavi status and access to the PAHO revolving fund. As no NGRV is yet on the market, we established hypothetical prices for iNGRV and oNGRV based on personal communications with vaccine developers and public health officials. oNGRV prices were generally set at the lowest LORV levels, while the prices of a standalone iNGRV and the iNGRV moiety included within a DTP-containing formulation (i.e., the incremental rotavirus vaccine-associated cost) were assumed to be approximately 1/3 and 2/3 lower per dose, respectively, than the lowest LORV prices [27]. Wastage, syringe and safety box costs and handling and transportation costs were taken into account as appropriate.

(iv) *Analyses of health and economic impact of iNGRV*. These analyses are described in detail in a full-length paper by Debellut et al. [27]. Briefly, we used a proportionate outcomes model, UNIVAC (version 1.4.16), to generate estimates of rotavirus disease events, intussusception disease events, and costs (vaccine program and healthcare costs) with and without rotavirus vaccination. We applied data from a metanalysis that provides efficacy data per under age five mortality stratum [16,29] to set efficacy values for LORVs, namely at 95%, 76%, and 45% in low-, medium-, and high-mortality settings, respectively. Regarding iNGRV, the absence of definitive efficacy data led us to explore scenarios in which three infant doses of iNGRV show efficacy similar to the previous LORV values or, alternatively, 100%, 83%, and 66% efficacy, again in low-, medium-, and high-mortality settings, respectively. For oNGRV, based on modelized Phase 2 efficacy results for RV3-BB [16], we assumed three doses, beginning with neonates, would have 97%, 80%, and 55% efficacy in those settings, respectively. We also included a sensitivity analysis in which oNGRV efficacy reached the higher figures postulated for iNGRV.

(v) *Determination of the feasibility and acceptability of immunization with iNGRV*. Informal discussions with global immunization and rotavirus disease experts revealed a widely held assumption that an iNGRV with similar efficacy to currently available oral vaccines may have little value due to strong preferences for oral over parenteral delivery. In fact, some suggested any oral rotavirus vaccine would be strongly preferred in LMICs even if the iNGRV offered substantially higher efficacy. It has also been suggested that co-administration of LORVs with moderately effective iNGRVs to improve overall rotavirus protection would be dismissed as too complicated, expensive, or both. As the ultimate validation of these assumptions—actual demand—could only occur if each became available as licensed products and all use cases were offered, an unlikely event, we decided to systematically query stakeholders in LMICs using our hypothetical use cases.

The NGRV feasibility and acceptability study involved individual, in-person interviews with national stakeholders [30] and healthcare providers [31] from Ghana, Malawi, Kenya, Peru, Sri Lanka, and Senegal. Countries were selected to represent a variety of socio-economic and epidemiological situations as well as eligibility for Gavi co-financing. National stakeholder interviews were conducted with individuals involved in immunization programs or policy-making and provider interviews with individuals currently administering vaccines at the primary healthcare level. Interviews in both study groups centered around a series of vaccine comparisons involving LORVs and hypothetical NGRVs (Table 1). Comparing two vaccines at a time, participants were asked to indicate which one they preferred based on information displayed in a visual aid on each of the vaccine’s known (for LORV) or assumed (for NGRV) attributes, featuring different presentations and efficacy for the NGRVs compared to LORV. The underlying rationales for vaccine preferences were elicited in open-ended questions.

Comparisons C1 and C2 with national stakeholders (Table 1) focused on the acceptability of a standalone iNGRV if available at significantly lower cost and assumed to have higher (iNGRV-H in C1) or similar (iNGRV-M in C2) efficacy compared to LORV. C3 and C4 examined the acceptability of co-administering iNGRV-M with LORV to enhance overall rotavirus protection. In C3, iNGRV-M is co-administered as a standalone formulation, whereas in C4 iNGRV-M is provided as part of a DTP-containing combination vaccine (iNGRV-DTP). C5 explored the perceived value of iNGRV-DTP compared to an equally effective LORV. C6 and C7 focused on the potential appeal of an oNGRV with a neonatal dose versus equally effective iNGRV options. Although following a similar guide, interviews with healthcare providers included fewer vaccine comparisons (Table 1) and focused more on delivery considerations.

(vi) *Elaboration of iNGRV demand forecasts for rotavirus vaccines*. The demand forecast model used a Monte Carlo simulation to forecast vaccine demand from 2020–2030 for a total of 107 countries, including countries eligible for Gavi support and LMICs that are not eligible [32]. Product preferences from national stakeholders were converted into a switch probability for use as the random variable in the simulation. This value represented a country’s probability of switching from a LORV product to a new NGRV product once introduced to the market. The switching probability was calculated as the weighted average of all product preference responses from each country’s national stakeholders. Each stakeholder was asked for their preference for either the current LORV product or a new NGRV product. Each stakeholder response was assigned a value of 1. This value was then multiplied by a weight set by their response and strength of preference. Weights of 1, 0.75, or 0.5 were applied if they chose a new NGRV product and their strength of preference was strong, moderate, or indifferent, respectively. Weights of 0.5, 0.25, or 0 were applied if they chose the current LORV product and their strength of preference was indifferent, moderate, or strong, respectively. The final switch probability was calculated for a country by summing all weighted values and dividing by their total number of stakeholders. A 100% switch probability represented a strong preference for the NGRV product from all stakeholders, whereas a 0% switch probability meant a strong preference for the LORV product. A 50% switch probability represented indifference between the two products.

Preferences collected from six countries in the feasibility and acceptability study were then extrapolated to other LMICs using a semi-supervised label spreading method based on similarities in World Bank Status, GDP per capita, and Gavi co-financing status in 2026. In addition to the product preferences expressed above, this model also relied on Dalberg country product preference analyses for LORVs before any NGRV introduction [33].

The Monte Carlo simulation was created for all comparison scenarios to model each country’s decision to switch to the NGRV product. In the introduction year, each country’s decision to continue using the LORV product or switch to the new NGRV product was made randomly based on their calculated switch probability. A country’s total demand was assigned to the chosen vaccine for each year from the introduction year through 2030. The total demand for each vaccine was then summed across all countries in the model for each year from 2020–2030. This simulation was repeated 10,000 times to determine the expected value for demand in 2030. Demand results across all 10,000 simulations are summarized with the arithmetic mean ± the standard error (SE) and 95% quantile interval. The 95% quantile interval was determined from the simulated demand distribution using the 0.025 and 0.975 quantiles.

## 4. Results

### 4.1. The Tangible Need to Prioritize among iNGRV Use Cases

Before undertaking any analyses, we first considered whether it was necessary to define and prioritize among specific iNGRV use cases, as it is of course possible that a single vaccine formulation may ultimately prove relevant for the various use cases discussed above. However, as illustrated in Table 2, determination of the primary use case (and necessary vaccine attributes) is actually necessary for efficient product and clinical development. For example, the clinical indication considered most important by the vaccine developer will dictate the objective of the pivotal clinical study, shaping the design and size of the clinical study program needed for licensure. Similarly, a high priority given to the low cost of goods (COGs) or combinability would point to the need to invest early to ensure it is possible to sufficiently optimize product manufacture or create specific formulations. Table 2 also highlights potential market implications of the specific theoretical advantage prioritized as reflected in the potential degree of interest that might be taken by certain global and national recommending bodies.

We, therefore, conducted two targeted analyses to see if it were possible to limit the number of iNGRV use cases to be analyzed in detail to those that seemed most promising.

### 4.2. Focus on Infant Vaccination, Rather Than Booster

The first analysis critically examined the case for an iNGRV booster dose, suggested it to be of special importance for high-morbidity settings [13,15]. This has largely been predicated on the observation that differences in calculated vaccine efficacy and effectiveness in the first versus second year of life can be as large as 40% [16,17]. It is tempting to suggest that the diminishing infant immune response to LORVs seen in high-morbidity settings is responsible for the decrease in calculated clinical efficacy [13] and could be restored with a booster dose.

To better evaluate this, we examined the modelized results of Burnett et al. [20], who had combined age-stratified LORV efficacy data with rotavirus mortality age-incidence rates to estimate the number of rotavirus deaths that could be averted by effective booster vaccination of LORV-immunized children in medium and high child mortality countries. Importantly, their calculations assume that infant coverage with rotavirus vaccine starting at 6 weeks of age is equal to DTP coverage in each country, regardless of the actual status of rotavirus vaccine introduction. Unfortunately, a missing piece of information is the kinetics of vaccine waning. Because calculations of vaccine efficacy are usually determined at very discrete intervals (e.g., 1 year, 2 years), though it is not likely that decreases in efficacy actually occur in an abrupt stepwise fashion, Burnett et al. [20] modeled multiple scenarios using the assumption that the pattern of waning is linear or, alternatively, logarithmic.

Furthermore, as the magnitude of the clinical benefit with boosting remains unknown, they developed an optimistic scenario in which vaccine efficacy in the second year of life was boosted above first year efficacy by 50% of the difference between vaccine efficacy in the first and second years of life. The results, extracted from [20], are depicted in Table 3. Under this scenario, a booster dose provided at 12 months of age could prevent 4–12% of the rotavirus deaths from occurring despite primary immunization with LORV. A booster at 9 months of age under the same scenarios only marginally increases those percentages (not shown).

The second aspect of our analysis looked for evidence to quantify the proportion of the observed vaccine waning that might be attributable to natural immunization of the unvaccinated children during the course of the LORV studies *(*Table 4), rather than to failure of the vaccine itself. In a careful dissection and reanalysis of a clinical efficacy study in Bangladesh (the PROVIDE study), Rogawski et al. [21] calculated vaccine efficacy against severe rotavirus disease by looking only at the subset of children (in the vaccine and control groups) who had no prior exposure to rotavirus as assessed by episodes of mild rotavirus disease. By comparing this value to that obtained by the usual method of all vaccinated versus all non-vaccinated children, the authors were able to offer an estimate of the impact of natural immunity in that setting. Assuming that the degree of natural immunity would be directly proportional to the background incidence of rotavirus disease, the authors then estimated the extent of natural immunity in five other settings where clinical trials have taken place. They were then able to model how much of the “waning” could, in fact, be attributable to increases in natural immunity (i.e., is “artifactual”) in each setting (Table 4).

These results suggested that an average of 40% of the decline in vaccine efficacy over the first year of life is due to natural immunization, not a secondary vaccine failure, but this may still overestimate the extent of vaccine waning. As noted in the editorial that accompanied the Rogawski et al. paper, undetected asymptomatic rotavirus infections likely providing additional natural immunity [22] are not factored into the calculations here. The combined contribution of both symptomatic and asymptomatic infection to improving the immunity of the unvaccinated group could thus easily account for more than half of the putative waning of LORV efficacy. This highlights the limited potential value of any rotavirus vaccine booster given late in the first year of life. It is noteworthy that the same considerations involving natural immunity might decrease the magnitude of some of the geographic differences in vaccine efficacy reported even within the first year of life. These results are consistent with the conclusions of a more recent mathematical model-based analysis focusing on the prevention of rotavirus hospitalizations. In that analysis, Pitzer et al. [34] predicted that an additional vaccine dose at 9 months of age would lead to very modest gains in vaccine impact. Taken together, these analyses indicated that our detailed exploration of an iNGRV value proposition should focus on its use in early infancy.

### 4.3. iNGRV Combination Vaccine Scenarios Should Focus on Potential Combinations with DTP-Hib-HepB and DTP-Hib-HepB-IPV, Not with IPV

As noted earlier, iNGRVs may be most valuable if combined with a vaccine already being routinely delivered in infancy. To further circumscribe, if possible, the number of scenarios to examine in our more detailed analyses, we reasoned that the ideal vaccine to combine with an iNGRV would be one projected to be available in large volumes at the approximate time of iNGRV availability and WHO prequalification (assumed as 2025 to 2027), and likely to remain in strong demand as an infant vaccine through 2030 and beyond.

**Figure 1:**
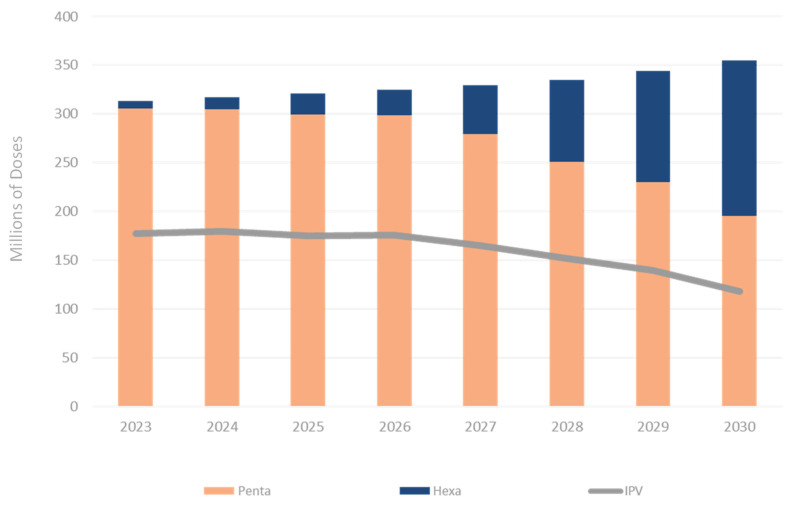
Estimated annual LMIC demand for DTP-pentavalent, DTP-hexavalent, and IPV in 2023 to 2030. Results are expressed in millions of doses. *Penta*: DTP-Hib-HepB; *Hexa*: DTP-Hib-HepB-IPV. See text for details.

While it was beyond the scope of this value proposition to examine the technical feasibility of specific antigen combinations, we made the *a priori* assumption that co-formulation of an iNGRV with multivalent pneumococcal conjugates already containing 10 or more separate components was highly unlikely. In addition, for this particular analysis, we excluded from consideration a variety of other potentially desirable combination vaccine partners for iNGRV, such as vaccines directed against other enteric pathogens, such as norovirus, enterotoxigenic *E. coli*, or *Shigella*, simply because those vaccines are not yet licensed or available and meaningful demand forecasts are not available. We, therefore, forecasted demand for LMICs from 2023–2030 for DTP-pentavalent, DTP-hexavalent, or IPV to determine which of those vaccines met the criteria of large volume and enduring demand. Modelized results were based on a number of assumptions about vaccine efficacy and attributes described in Section 3. Figure 1 below shows a base scenario of four DTP-hexavalent suppliers and encompasses those LMICs that have DTP-pentavalent and IPV currently in their immunization schedules.

The Figure suggests a markedly diminishing market for standalone IPV by 2030 (only a subset of which would be delivered to young infants alongside a DTP-containing vaccine), a diminishing but more substantial market for DTP-pentavalent, and a burgeoning DTP-hexavalent market. These results may indicate that either DTP-containing vaccine could be an attractive iNGRV “partner” for several years.

These initial analyses allowed us to focus our subsequent impact and cost-effectiveness analyses and feasibility and acceptability study on a limited number of use cases for administering iNGRV to young infants, whether as a standalone three-dose vaccine or combined with DTP-pentavalent or DTP-hexavalent (the latter two subsequently referred in the rest of this article as “DTP-containing formulations”). As there is evidence of synergistic effects with certain vaccines acting through distinct immunological mechanisms when co-administered or given as a heterologous prime-boost [35], we also assessed the scenarios of a moderately effective iNGRV given alongside LORV or oNGRV. These results were then used to forecast demand in a large group of LMICs using the same use cases.

### 4.4. Potential Impact and Cost-Effectiveness of Different Use Cases of iNGRV

Using the proportionate outcomes model UNIVAC (version 1.4.16), we estimated the projected impact of LORVs, iNGRV, and oNGRV with different attributes and under various use cases (see Section 3) on several health and economic outcomes (Table 5). Compared to no rotavirus vaccination, over a 10-year period LORVs are projected to avert approximately 550,000 rotavirus gastroenteritis deaths, 10.2 million hospitalizations, and 250 million cases, resulting in a savings of 14.5 million DALYs. An iNGRV with substantially higher efficacy would avert an additional 200,000 rotavirus deaths, 3 million hospitalizations, and 70 million rotavirus cases, preventing an additional 5 million DALYs.

Differences between standalone and combination formulations of iNGRV were readily apparent when economic outcomes were examined. An iNGRV-DTP-containing combination, in particular, would have the lowest overall program cost (US$3 billion) of all options and be cost-saving compared to no rotavirus vaccination. Strikingly, even if the efficacy of the iNGRV-DTP-containing formulation were determined to be no greater than that of LORV, the combination vaccine would remain more affordable and cost-effective than all other options. A standalone, high-efficacy iNGRV, while incurring considerably greater program costs than the combination, would provide maximal benefits and would still be cost-effective in 84% of LMICs at a willingness-to-pay threshold of 0.5 times the national GDP per capita in each country (data not shown). It would be more cost-effective than oNGRV, which in turn would be more cost-effective than the most favorable LORV, ROTAVAC. Importantly, the latter is still cost-effective in 67% of LMICs at this threshold [27].

The results shown here (and more extensively in [27]) would support a positive public health value proposition for an iNGRV, especially one formulated as part of a larger DTP-containing combination. It also highlights that, while co-administration scenarios of LORV with a moderately-effective iNGRV would result in high benefits, they are generally much less cost-effective unless iNGRV is provided as part of a larger DTP-combination vaccine. Much attention has been focused on the value of potential enhancements in the clinical efficacy of an NGRV, whether parenterally or orally administered, but it is noteworthy how much the ability to include an iNGRV as part of a larger DTP-containing combination would reduce costs, even if it were no more efficacious than LORVs. This is a function of the relatively low cost of a bulk rotavirus vaccine moiety along with no incremental costs of delivery.

### 4.5. Product Preferences among Country Stakeholders in LMICs

Eight to fifteen national stakeholders from each country (see Methods) agreed to be interviewed (*n* = 71 total), more than half of whom served on national immunization advisory groups. A similar number of healthcare providers in each country were interviewed (*n* = 64), with the exception of Sri Lanka where LORV has not yet been introduced.

### 4.6. High-Level Findings from National Stakeholder Interviews

**Figure 2:**
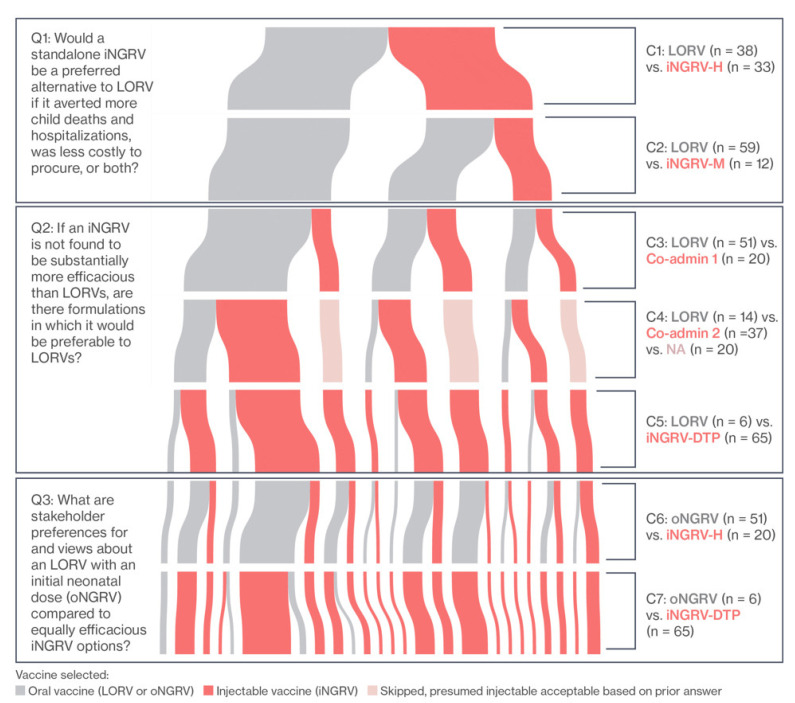
National stakeholder preferences for existing and next-generation rotavirus vaccines. Results from fixed-choice questions in mixed method interviews with 71 national stakeholders from Ghana, Kenya, Malawi, Peru, Senegal, and Sri Lanka. *C1* through *C7* refer to Comparison 1 through Comparison 7. *LORV*: live, oral rotavirus vaccines; *iNGRV-M* and *iNGRV-H*: injectable next-generation rotavirus vaccines, with *M*oderate and *H*igh efficacy, respectively; *iNGRV-DTP*: injectable next-generation rotavirus vaccine co-formulated with DTP-Hib-HepB or DTP-Hib-HepB-IPV; *oNGRV*: oral next-generation rotavirus vaccine. See text for details. Figure reproduced from [30].

As illustrated in Figure 2, oral rotavirus vaccines were generally preferred over the standalone parenteral versions. Concerns about injection fatigue among both parents and healthcare providers were the most frequently cited reason by national stakeholders for preferring the oral option over iNGRV, followed by additional cold chain requirements and operational complexities. More generally, requiring “nothing new” was a strong theme expressed by those who preferred LORV to iNGRV, despite its higher cost per fully immunized child (C1 and C2). For stakeholders preferring oNGRV to iNGRV (C6), the possibility of providing early protection from rotavirus to infants was an additional attraction, again despite its higher cost compared to iNGRV. Nonetheless, even while perceiving the same disadvantages with injectables, close to half of the national stakeholders preferred iNGRV-H over LORV (C1), explaining that its higher efficacy outweighed its shortcomings.

A pronounced and sustained shift in the preference pattern in favor of iNGRV occurred when it was presented as part of a DTP-containing vaccine—i.e., DTP-pentavalent or DTP-hexavalent (iNGRV-DTP) in C4, C5, and C7. This shift was seen for iNGRV-DTP in comparison with LORV when vaccine efficacy was set at LORV levels, and for iNGRV-DTP over oNGRV when efficacies were set at a higher level. Even co-administration of iNGRV-DTP with LORV to improve overall rotavirus vaccine protection was preferred over LORV alone, despite the higher cost and complexity.

Taken together these results indicate that, while greater rotavirus protection remained important for a number of national stakeholders, the overwhelming preference was to avoid additional vaccine administrations, oral or injectable. As such, interest in a standalone iNGRV was mixed, despite the potential higher efficacy. In contrast, if iNGRV were included within a DTP-containing combination vaccine, it would become the dominant choice, even if it did not present any efficacy advantage over the oral vaccine. This enthusiasm was only somewhat tempered if such a combination were available from a single supplier. More detailed results and discussion are presented in Price et al. [30].

### 4.7. High-Level Findings from Healthcare Provider Interviews

Of the 64 providers asked to choose between an oral versus an injectable rotavirus vaccine (C1a and C2a in Table 1), 58 and 59 chose LORV (C1a) and the neonatal oNGRV (C2a), respectively, due primarily to injection reluctance and perceived ease of oral delivery. Results were more evenly split in a direct comparison between the two oral vaccine options (C3a in Table 1), 37 individuals preferring the neonatal oNGRV versus 27 for the current LORV. While the prospect of earlier protection was frequently emphasized among those who preferred oNGRV, the success of current LORV programs was often cited by those who preferred LORV. Interestingly, roughly half who chose oNGRV foresaw, nonetheless, significant logistical challenges including integration with maternal and child health programs and the need for additional maternal education.

Challenges in co-administering standalone iNGRV with LORV and concerns about delivering an iNGRV-DTP combination vaccine were explored in two open-ended questions. Roughly half of the vaccinators indicated one or more difficulties administering both LORV and iNGRV, including educating mothers about the vaccine schedule in addition to various operational and injection concerns. While an overwhelming majority expressed enthusiasm for an iNGRV-DTP, 18 individuals described concerns stemming from mothers’ complaints about pain for the child from the existing DTP-containing vaccine or concerns about the safety of an iNGRV-DTP for immature immune systems.

These results, presented in more detail by Mooney et al. [31], suggest that introduction of any NGRV would face specific challenges in some settings and highlight the need for more education, training, and tailored messages for healthcare providers.

## 5. Projected Demand for iNGRVs

The product preferences expressed by national stakeholders were then used to inform a product demand forecast model based on Linksbridge’s Global Vaccine Market Model demand and supply forecasts [25]. This analysis assumes that aggregated national stakeholder responses accurately represent vaccine preference given a choice between LORV and NGRV products and that the policy decisions will be made in line with the specified preferences. See *Methods* for details as to how these data were used to project demand.

Under the assumption that there are no supply limitations, and in the absence of oNGRV, mean demand for a high efficacy iNGRV was estimated at 50M (±100K SE) 3-dose courses by 2030, ranging from 30M–69M in the 95% quantile interval. This mean demand represented 42% of the total rotavirus vaccine market in the 107 countries. Demand for an iNGRV with similar efficacy to LORVs reached an average of 21M (±86K SE) 3-dose courses, ranging from 7.4M–40M in the 95% quantile interval. This mean demand could increase by 50%, however, if iNGRV were co-administered with LORVs.

In contrast, under the assumption that there are multiple suppliers of iNGRV-DTP, even if iNGRV efficacy were only similar to that of LORV, mean demand for that formulation was estimated at 99M (±68K SE) 3-dose courses, ranging from 82M–109M in the 95% quantile interval. In this scenario, the mean demand took up 84% of the rotavirus vaccine market in 2030. Finally, if both a high efficacy oNGRV and high efficacy iNGRV-DTP were on the market, the latter’s mean demand was estimated at 86M (±87K SE) 3-dose courses, ranging from 68M–99M in the 95% quantile interval. This mean demand represented 73% of the rotavirus vaccine market in 2030.

## 6. Discussion

The analyses presented here suggest there is a compelling public health value proposition for interventions that will improve the impact of rotavirus vaccination in young infants, rather than complement existing LORVs with a booster dose at 9 or 12 months. The results point to two ways an iNGRV could contribute to such improvements.

First, an iNGRV could enhance vaccine efficacy in young infants, either by possessing intrinsically higher potency (and thus replacing LORVs) or by eliciting a synergistic clinical efficacy response upon co-administration with the existing LORVs. The results described here suggest there would be significant health and economic benefits of higher efficacy and thus, provide additional evidence in favor of the ongoing development of parenterally administered NGRVs.

Second, our analyses highlight that an iNGRV may significantly improve rotavirus vaccination coverage simply by being formulated as part of a DTP-containing vaccine. Whether delivered alone or co-administered with LORV, iNGRV-DTP emerged as the most cost-effective option compared both to existing products and oNGRVs [28]. It was also the option most preferred by country stakeholders against all other options [31], and, by extension, to likely have the greatest market demand, whether its efficacy was similar or superior to that of existing products. The combined findings from all three major analyses—the health impact and economic analysis, the feasibility and acceptability study, and the demand forecast—underscore a need for funders and manufacturers to consider prioritizing the necessary iNGRV compatibility and combinability studies with DTP-pentavalent and DTP-hexavalent, even if iNGRV efficacy ends up being no greater than that of existing LORVs [8]. Given the considerable effort, investment, and timelines typically required for combination vaccine development, it could make sense to initiate such studies now while the candidates remain at least a few years away from licensure.

As there are multiple valuable use cases involving an iNGRV whose efficacy is similar to that of current LORVs, we further suggest that Phase 3 iNGRV clinical study designs should incorporate demonstration of clinical non-inferiority of iNGRV to LORV as the primary objective, with the demonstration of superiority as a secondary objective. We also provide evidence that an oNGRV following a neonatal schedule would appeal to national stakeholders and many healthcare providers alike and would likely find a favorable market.

Finally, we believe that the focus and analyses undertaken within this iNGRV value proposition represent a departure from many of the vaccine-centered investment case efforts available to date. A recent review of 19 such cases developed over the past 35 years [36] noted that only five focused on vaccine candidates in the pipeline, with the rest covering already licensed and introduced vaccines. Only a handful of the 19 were published as complete journal articles, with the remainder largely discovered on agency websites. Almost all described the burden of disease and highlighted the potential health economic impact of the vaccines, with a predominant focus on the impact on mortality. Most discuss “health system capacity”, but only one-third considered how the interventions might “align with target audience goals”. The authors also note that many of the documents are explicitly intended for advocacy, rather than attempting to be critical, objective analyses, and it is not always clear where their funding support comes from [36].

In contrast, we provided a comprehensive, critical focus on a yet-to-be-licensed class of vaccines (iNGRVs), their potential benefits on both morbidity and mortality, as well as economic impact, and explicitly sought to frame this in the context of the expressed interests of LMIC stakeholders. Regarding the latter, we found that the inclusion of both national stakeholders and healthcare providers in the feasibility and acceptability study allowed us to glean insights and preferences, especially on the programmatic side, which are not necessarily identical to those of global experts. We believe that the multi-stage analytic process—evidence review, health impact and cost-effectiveness study, feasibility and acceptability study, and demand forecasting—combined with the focus on multiple attributes and inclusion of mixed data types, helped produce a complete and credible value proposition.

In conclusion, depending on the specific use cases and attributes, there appears to be a compelling public health value proposition for the development and introduction of iNGRVs for young infants. This iNGRV value proposition thus adds to a growing body of work on national prioritization and decision-making for new vaccine introductions [37] as well as to efforts to ensure that products brought to market respond to LMIC needs [4].

## Figures and Tables

**Table 1:** Comparisons Used in LMIC Stakeholder Interviews of LORVs *vs*. Hypothetical NGRVs.

Comparisons with National Stakeholders				Comparisons with Healthcare Providers			
C1	LORV	*vs.*	iNGRV-H	C1a	LORV	*vs.*	iNGRV
C2	LORV	*vs.*	iNGRV-M	C2a	oNGRV	*vs.*	iNGRV
C3	LORV	*vs.*	Co-admin 1	C3a	LORV	*vs.*	oNGRV
C4	LORV	*vs.*	Co-admin 2				
C5	LORV	*vs.*	iNGRV-DTP				
C6	oNGRV	*vs.*	iNGRV-H				
C7	oNGRV	*vs.*	iNGRV-DTP				

Abbreviations: iNGRV-H—standalone iNGRV assumed to have substantially higher efficacy than LORV; iNGRV-M—standalone iNGRV assumed to have moderate efficacy, similar to LORV; iNGRV-DTP—iNGRV-M provided through an iNGRV-DTP-containing vaccine; Co-admin 1—LORV and iNGRV-M both given to achieve substantially higher efficacy; Co-admin 2—LORV and iNGRV-DTP both given to achieve substantially higher efficacy; oNGRV—next generation oral vaccine initiated with a birth dose. Note: vaccine efficacy information was not provided to healthcare providers.

**Table 2:** Implications of deciding on the primary theoretical advantage of iNGRV.

Primary Theoretical Advantage of iNGRV over LORVs	Clinical Endpoint Needed	Chemistry, Manufacturing, and Controls (CMC) Implications	Recommending Body/Market Implications
**Higher vaccine efficacy in high-morbidity settings**	Demonstrate NGRV’s vaccine efficacy (VE) superiority to LORV.	n/a	Strong selling point to WHO/Strategic Advisory Group of Experts (SAGE) and low-income, high-morbidity settings but perhaps not to lower morbidity middle income countries (MICs).
**Lower Cost of Goods (COGs)/dose**	Demonstrate VE non-inferiority to LORV.	Focus on technologies to minimize COGs.	If prices lower than LORV, an NGRV would be attractive to Gavi and LMICs supporting their own vaccine costs. Not clear if low-income countries currently supported by Gavi would see this as a sufficiently compelling reason to choose NGRV over LORVs, nor whether lower COGs would translate into prices sufficiently low enough to attract MICs that have not yet introduced rotavirus vaccine.
**Co-administration with LORVs or as a boost to counteract reduced vaccine impact over time**	No need to demonstrate VE after primary series but must show enhanced VE compared to LORV alone upon co-administration or boost.	n/a	COGs advantage over LORVs lost; unclear if preventing incremental late disease sufficiently impactful to affect global recommendations or national uptake.
**Can be combined with DTP-containing vaccines or IPV**	Demonstrate VE non-inferiority to LORV, plus immunological non-inferiority in the combination form and non-interference with other antigens.	Major investment needed; physicochemical compatibility efforts prioritized; need to reduce iNGRV dosage volume and potentially interfering excipients.	Delayed time to market compared to a standalone product, but if only one manufacturer is successful might allow it to dominate DTP-containing combination vaccine field.
**No vaccine-induced intussusception**	Demonstrate VE non-inferiority to LORV. (Impossible to demonstrate lack of heightened risk of intussusception pre-licensure.)	n/a	Unclear if vaccine-induced intussusception observed primarily in low-mortality countries is a barrier to uptake of LORVs in other settings.

**Table 3:** Rotavirus deaths estimated preventable by a highly effective vaccine booster dose.

Region	Linear Waning	Logarithmic Waning
**Deaths occurring annually despite high oral rotavirus vaccine coverage, without a booster.**		
Africa	62,466	62,382
Southeast Asia	28,507	27,838
**Deaths preventable by 12-month booster increasing vaccine efficacy by 50%**		
Africa	2658 (4.3%) *	4035 (6.5%) *
Southeast Asia	2153 (7.6%) *	3269 (11.7%) *

Modeled estimates based on rotavirus mortality by age, assumption of 65% and 45% vaccine efficacy for LORV in first and second years of life, respectively, in the absence of a booster, and that booster increases second year efficacy above that seen in the first year by 50% of the difference between first and second year efficacies. These examples assumed efficacy wanes linearly or logarithmically. * Parentheses express preventable deaths as a percentage of deaths occurring annually in the absence of a booster. Data from Burnett et al. [20].

**Table 4:** Proportion of LORV efficacy “waning” potentially attributable to natural immunization of unvaccinated children.

Study Site	LORV Efficacy Waning (% Decrease between Reported 1st and 2nd Year Vaccine Efficacies *)	How Much Higher Second Year Efficacy Should Be (Excludes from Efficacy Calculations Unvaccinated Children Likely Naturally Immunized by Mildly Symptomatic Rotavirus Infections) *	Percentage of “Waning” that Appears Artifactual **
South Africa	36.9%	5.8%	16%
Ghana	35.1%	10%	28%
Bangladesh	42.2%	15.5%	37%
Mali	23.7%	14.8%	62% *
Malawi	31.8%	18%	57% *
**Average**	**33.9%**	**12.8%**	40% *

* Data extracted from Rogawski et al. [21]; see text for details. ** The percentage, representing the estimated contribution of natural immunity, was calculated by present authors by dividing each value in the second column by the corresponding value in the first column.

**Table 5:** Impact and cost-effectiveness results per vaccination use case for all LMICs over 10 years starting in 2025 (use cases ordered by net cost).

Vaccine(s)	Averted RVGE Cases	Averted RVGE Hospitalizations	Averted RVGE Deaths	Additional IS Deaths	Averted DALYs (Discounted)	Vaccine Program Costs	Averted Healthcare Costs	Net Cost	Cost-Effectiveness Ratio
iNGRV-DTP	322,134,000	13,053,000	754,000	0	19,643,000	1,393,077,000	2,716,684,000	−1,323,607,000	Cost-saving
iNGRV-DTP-M	256,731,000	10,424,000	573,000	0	14,991,000	1,393,077,000	2,332,835,000	−939,759,000	Cost-saving
iNGRV	322,134,000	13,053,000	754,000	0	19,643,000	8,250,914,000	2,716,684,000	5,534,230,000	282
iNGRV-M	256,731,000	10,424,000	573,000	0	14,991,000	8,250,914,000	2,332,835,000	5,918,079,000	395
oNGRV or oNGRV-H	288,677,000 328,462,000	11,713,000 13,316,000	636,000 748,000	470	16,650,000 19,510,000	9,440,011,000	2,580,877,000 2,812,059,000	6,627,952,000 6,859,134,000	340 412
ROTAVAC ROTASIIL	251,184,000	10,198,000	556,000	1530	14,524,000	9,375,359,000 10,403,578,000	2,294,338,000	7,081,020,000 8,109,240,000	488 558
iNGRV-DTP with oNGRV, ROTAVAC, or ROTASIIL	322,134,000–328,462,000	13,053,000–13,316,000	748,000–754,000	470–1530	19,510,000–19,604,000	10,833,088,000– 11,796,655,000	2,714,128,000– 2,812,059,000	8,021,029,000–9,082,527,000	411–463
iNGRV with oNGRV, ROTAVAC, or ROTASIIL	322,134,000–328,462,000	13,053,000–13,316,000	748,000–754,000	470–1530	19,510,000–19,604,000	17,690,925,000– 18,654,492,000	2,714,128,000–2,812,059,000	14,878,866,000–15,940,364,000	763–813
ROTARIX	251,184,000	10,198,000	556,000	1530	14,524,000	24,075,203,000	2,294,338,000	21,780,865,000	1500
iNGRV-DTP or iNGRV with ROTARIX	322,134,000	13,053,000	754,000	1530	19,604,000	25,468,279,000 32,326,116,000	2,714,128,000	22,754,152,000 29,611,989,000	1161 1510

RVGE: Rotavirus gastroenteritis. IS: Intussusception. DALY: Disability-Adjusted Life Years. iNGRV-DTP: iNGRV co-formulated with DTP-pentavalent or DTP-hexavalent. Suffixes “-M” and “-H” refer to moderate- or high-efficacy versions of the vaccine (see text). Data extracted from [28]. Cost-effectiveness ratio for each vaccination use case = Net cost/Averted DALY.
